# Influences of dietary protein sources and crude protein levels on intracellular free amino acid profile in the *longissimus dorsi* muscle of finishing gilts

**DOI:** 10.1186/s40104-015-0052-x

**Published:** 2015-12-18

**Authors:** Chunfu Qin, Ping Huang, Kai Qiu, Wenjuan Sun, Ling Xu, Xin Zhang, Jingdong Yin

**Affiliations:** State Key Lab of Animal Nutrition, College of Animal Science & Technology, China Agricultural University, Beijing, 100193 China; Chongqing Academy of Animal Science, Rongchang, Chongqing, 450023 China

**Keywords:** Dietary protein source, Finishing gilt, Muscle free amino acids, Nitrogen efficiency, Performance, Pork quality

## Abstract

**Background:**

The current study was carried out to determine effects of dietary protein source and crude protein (CP) level on carcass characteristics, meat quality, and muscle amino acid (AA) profile in finishing gilts. The experiment was designed as a 2 × 2 factorial arrangement with two sources of dietary proteins (cottonseed meal, CSM vs. soybean meal, SBM) and two levels of CP (12 % vs. 14 %, as-fed basis). Seventy-two crossbred gilts (89.5 ± 0.9 kg) were allotted to one of four dietary treatments in a randomized complete block design for a period of 28 d. All diets were formulated to be isoenergetic and similar concentrations of standardized ileal digestible essential AA covering the nutrient requirements of pigs.

**Results:**

Growth, carcass characteristics and meat quality were not affected by dietary protein source nor crude protein level (*P* > 0.10) except that average daily feed intake was increased by CSM diets (*P* = 0.03). Gilts offered reduced protein diets had lower muscle pH_45min_ (*P* < 0.05). Neither dietary protein source nor crude protein level influenced N deposition. However, reduced protein diets decreased N intake, N excretion, and serum urea nitrogen content, whilst improved N efficiency (*P* < 0.01). CSM diets increased N intake (*P* = 0.04), but did not depress N efficiency. The concentrations of phenylalanine, tryptophan, cysteine and tyrosine (*P* < 0.05) of the *longissimus* muscle were decreased when gilts offered CSM diets, while muscle intracellular free valine concentration was increased (*P* = 0.03). The gilts offered reduced protein diets had greater intracellular concentrations of free methionine, lysine, and total AA in muscle (*P* < 0.05).

**Conclusion:**

These results suggest that CSM could replace SBM as a primary protein source in finishing pig diets in terms of performance, N efficiency, carcass characteristics, and meat quality, but decrease the concentrations of muscle specific AA. Furthermore, the reduced protein diet played an important role in increasing muscle intracellular concentrations of specific free amino acids (FAA), and in reducing the relative ratios of specific FAA to lysine in *longissimus dorsi* muscle of pig, whose biological meaning needs further studies.

**Electronic supplementary material:**

The online version of this article (doi:10.1186/s40104-015-0052-x) contains supplementary material, which is available to authorized users.

## Background

In modern livestock production, producers wish to reduce N excretion, whilst minimizing production costs and improving productivity [1]. Soybean meal (SBM) has been the primary protein source in swine diet. However, the increasing demand for SBM raises the price of SBM dramatically in recent years because of the global enlargement of livestock production [2]. Therefore, there has been considerable interest in reducing SBM use in the feed industry.

Cottonseed meal (CSM) is more economical than SBM and frequently used in swine diets because of its high protein content [2]. However, CSM containing gossypol, which is a phenolic aldehyde and recognized as an anti-nutrient factor, limits CSM use in animal diets. Gossypol damages the capabilities of digestion, reproduction, etc., in humans and monogastric animals [3]. A recent study suggested that pig performance was not hampered if the gossypol in CSM-based diets was detoxified effectively [4]. However, the effects of total replacement of SBM with CSM on carcass characteristics, meat quality, and muscle amino acid (AA) profile of pigs remain undefined.

Feeding reduced protein diets is another way to reduce the reliance on SBM and simultaneously cut down N excretion. It did not hamper pig performance when the low protein diets had been judiciously supplemented with crystalline essential amino acids (EAA) [5, 6]. However, effects of reduced protein diets on carcass characteristics, meat quality, and especially muscle AA profile have not been examined.

Therefore, the present study aimed to investigate influence of dietary protein sources (CSM vs. SBM) and crude protein (CP) levels (12 % vs. 14 %) on carcass characteristics, meat quality, and AA profile of the *longissimus* muscle (LM) in finishing pigs.

## Methods

The experiment was carried out in accordance with the Chinese Guidelines for Animal Welfare and Experimental Protocol, and approved by the Animal Care and Use Committee of China Agricultural University.

### Animals and housing

Seventy-two crossbred gilts (Duroc × Landrace × Yorkshire) with an initial body weight (BW) of 89.5 ± 0.9 kg were selected from a commercial herd. All pigs were housed in the same building with slatted floors in Fengning Experimental Farm (Fengning County, Hebei Province, China). Gilts were randomly assigned by BW to one of four dietary treatments. There were three gilts per pen and six replicate pens per treatment in a randomized complete block design. All pens were equipped with a nipple waterer and a feeder. Feed and water were offered *ad libitum* throughout the period of the study. A commercial diet was fed for 3 d during the acclimation period. The feeding trial was lasted for 28 d. The floor was cleaned twice every day. Average daily feed intake was registered per pen weekly. All animals were weighed at the beginning and the end of the trial.

### Experimental diets

A 2 × 2 factorial arrangement with two sources of protein (SBM vs. CSM) and two levels of crude protein (12 % CP vs. 14 % CP as-fed basis) constituted the four dietary treatments (named CSM12, CSM14, SBM12, and SBM14, respectively). Cottonseed meal used in the present study was solvent-extracted degossypolized, with the residual free gossypol content at 63 mg/kg. Considering maximum supplementation of CSM in diets was 13.72 % (CSM14), the residual free gossypol content was below the maximal amount (60 mg/kg) allowed in the diet of growing-finishing pigs in China [7]. Ingredient composition and nutrient content of the experimental diets are shown in Table 1.

**Table 1:** Ingredient and nutrient content of diets, as-fed %

Item	CSM12^a^	CSM14^a^	SBM12^a^	SBM14^a^
Ingredients				
Corn	83.19	74.75	86.02	79.90
Wheat bran	3.30	5.40	1.75	2.70
Soybean meal	-	-	8.86	14.60
Cottonseed meal	8.15	13.72	-	-
Soybean oil	1.60	2.77	-	-
Limestone	1.06	1.15	0.98	1.04
Dicalcium phosphate	0.48	0.30	0.60	0.45
Sodium chloride	0.35	0.35	0.35	0.35
L-Lysine HCl	0.61	0.53	0.44	0.26
L-Threonine	0.21	0.17	0.15	0.07
L-Tryptophane	0.06	0.04	0.05	0.02
DL-Methionine	0.10	0.06	0.08	0.03
L-Valine	0.12	0.05	0.06	-
L-Isoleucine	0.17	0.13	0.08	-
L-Histidine	0.02	-	-	-
Choline chloride (50 %)	0.08	0.08	0.08	0.08
Vitamin-mineral premix^b^	0.50	0.50	0.50	0.50
Analyzed nutrient composition^c^				
Gross energy, Mcal/kg	3.86	3.95	3.78	3.79
Crude protein	11.82	13.88	12.31	14.34
Arginine	0.85	1.17	0.67	0.75
Histidine	0.32	0.39	0.34	0.37
Isoleucine	0.36	0.43	0.46	0.51
Leucine	1.15	1.24	1.33	1.39
Lysine	1.00	0.97	0.86	0.82
Methionine	0.33	0.30	0.28	0.28
Phenylalanine	0.55	0.67	0.58	0.63
Threonine	0.58	0.59	0.57	0.55
Tryptophan	0.15	0.16	0.15	0.16
Valine	0.68	0.72	0.69	0.67
Sulfur-containing amino acids^d^	0.62	0.60	0.54	0.57
Calculated nutrient composition^e^				
Metabolizable energy, Mcal/kg	3.30	3.30	3.30	3.30
Calcium	0.52	0.52	0.52	0.52
STTD phosphorus^f^	0.21	0.21	0.21	0.21

^a^CMS12, cottonseed meal as protein source, 12 % crude protein level; CMS14, cottonseed meal as protein source, 14 % crude protein level; SBM12, soybean meal as protein source, 12 % crude protein level; SBM14, soybean meal as protein source, 14 % crude protein level

^b^The vitamin-mineral premix provided the following quantities of vitamins and micro minerals per kilogram of complete diet: vitamin A as retinyl acetate, 4,000 IU; vitamin D_3_ as cholecalciferol, 1,000 IU, vitamin E as DL-alpha tocopheryl acetate, 10 IU; vitamin K_3_ as menadione nicotinamide bisulfite, 1.25 mg; thiamine as thiamine mononitrate, 0.5 mg; riboflavin, 2.1 mg; pyridoxine as pyridoxine hydrocloride, 1 mg; vitamin B_12_, 0.007 mg; D-pantothenic acid as D-calcium pantothenate, 6 mg; niacin as nicotinamide and nicotinic acid, 12 mg; folic acid, 0.25 mg; biotin, 0.02 mg; Cu, 10 mg as copper sulfate; Fe, 75 mg as iron sulfate; I, 0.025 mg as potassium iodate; Mn, 10 mg as manganese sulfate; Se, 0.02 mg as sodium selenite; and Zn, 45 mg as zinc oxide

^c^Analytical results obtained according to AOAC [17]

^d^Sum of methionine and cysteine

^e^Calculated values according to NRC [8]

^f^STTD = standardized total tract digestible

Experimental diets were formulated on the calculated AA contents, which were obtained from the analyzed AA contents multiplying by the standardized ileal digestibility (SID) coefficients for the corresponding ingredients [8]. All diets were supplemented with crystalline EAA to obtain the same concentrations of SID EAA to meet NRC [8] nutrient recommendations of pigs weighting 75 to 100 kg. In brief, all diets were equal in metabolizable energy (ME) (3.30 Mcal/kg) and in the supplies of SID lysine (0.73 %), tryptophan (0.13 %), threonine (0.46 %), and sulfur-containing amino acids (SAA, 0.42 %), as well as total calcium (0.52 %) and standardized total tract digestible (STTD) phosphorus (0.21 %). The remaining of SID EAA were formulated to meet or exceed NRC [8] nutrient recommendations. Feeds were sampled and stored at 4 °C for chemical analysis.

### Slaughter procedure and sample harvest

At the end of the trial, gilts with the average final BW from each pen were selected, after 12 h-fasting but free access to water, these pigs were transported to a local abattoir (30 min). After at least 4 h rest, an 8 mL of blood was sampled from each pig by vena cava puncture using a 9 mL clot activator tube (Greiner Bio-One GmbH, Kremsmunster, Austria). Serum were collected by centrifugation at 3,000 × g for 15 min within 2 h after collection and stored at −20 °C until analysis. After blood sampling, the gilts were electrically stunned, exsanguinated and eviscerated according to standard commercial procedure. The carcass was split down the center of the vertebral column, on the left half of each carcass, about 5 g of LM between the ninth and 10th ribs were sampled and stored at −80 °C for measurements of intramuscular fat content (IMF), AA profile, and intracellular FAA content.

After 24 h postmortem, LM on the left half of each carcass between the 10^th^ and 12^th^ ribs were sampled for analysis of meat quality.

### Measurement of carcass characteristics and meat quality

All the parameters were measured on the left side of each carcass, including HCW, back fat depth of the 10^th^ rib and the last rib back fat depth, and the LM area (LMA): LMA (cm^2^) = loin eye height (cm) × width (cm) × 0.7 (Eq.1). Before carcass chilling, hot carcass weight (HCW) was recorded within 45 min of postmortem. Dressing percentage for an individual animal was represented as a ratio of HCW to the live weight. Subjective marbling score of LM was conducted on the cut surface at the intercostal space between the 10^th^ and 11^th^ ribs [9, 10]. A 10-piont scale (1 = devoid, 10 = abundant marbling) was applied to evaluate the subjective marbling score according to NPPC [11] guidelines.

Muscle color, including L* (lightness), a* (redness), and b* (yellowness), was measured at 24 h postmortem at the level of the last rib on a cross section of the *longissimus thoracis* muscle using a Minolta chromameter (CR-410, Konica minota, Tokyo, Japan) after exposing the surface to the air for 30 min at 4 °C.

Drip loss and cooking loss were measured as described previously [12, 13].

At 45 min postmortem, an incision was made on the LM at the eighth *thoracic* vertebra and initial muscle pH_45min_ was detected with a SPK pH meter (pH-star, DK2730, Herlev, Denmark). Meanwhile, the pH_24 h_ was measured 24 h postmortem in the chilling room.

The shear force (kg) of LM was determined as modified from that of Ciobanu et al. [14]. Loin sections were collected from carcasses after 24 h postmortem and 10 cylindrical pork core samples (10 mm diameter × 10 mm length) from each meat section, then were cut parallel to the fiber orientation using a cylindrical cutter [15]. Peak shear force was determined by using a digital-display-muscle tenderness meter (C-LM3B, Tenovo, Harbin, China) [16].

### Chemical analysis

Feed ingredients and experimental diets were analyzed for dry matter, CP, phosphorus, and gross energy according to the procedures of the AOAC [17]. Samples were ground to pass a 1 mm screen using a lab mill before analysis. The contents of free gossypol in cottonseed meal [18] and the concentrations of AA in feed [19] were analyzed as described previously using HPLC (L-8900 AA Analyzer, Hitachi, Tokyo, Japan). For measurement of muscular AA profile, meat samples were vacuum freeze-dried and then ground in liquid nitrogen. In prior to HPLC analysis, three aliquots of the freeze-dried powder were submitted to alkaline hydrolysis, oxidation hydrolysis, and acid hydrolysis for analysis of tryptophan, SAA, and the rests of AA, respectively. The concentration of serum urea nitrogen (SUN) was assayed using a commercial kit according to the manufacturer’s instruction (C013-1, NJJC, Nanjing, China). Intramuscular fat content in LM was determined by ether extraction [20]. Intracellular FAA concentration in muscle was determined with the modification from that of Li et al. [21] using UPLC-MS/MS system (5500 QTRAP, Applied Biosystems, Life Technology, Forrest City, CA) equipped with a BEH T3 column (2.1 mm × 100 mm, 1.7 μm). Briefly, about 300 mg samples were homogenized in 500 μL water with 500 μL methanol (1:1, v:v). The homogenate was incubated at 4 °C for 30 min and then centrifuged at 10,000 × *g* for 10 min. Supernatant was 20-fold diluted in water with acetonitrile (25:75, v:v), then the mixture was filtered through glass wool for UPLC-MS/MS determination.

### Estimation of protein deposition, and N balance

Daily body protein deposition (Pd) (g/d) was estimated from BW by the equation Pd (g/day, gilts) = 137 × [0.7066 + (0.013289 × BW) – (0.00013120 × BW^2^) + (2.8627 × 10^−7^ × BW^3^)] (Eq. 2) [8].

Total N intake was computed as: total feed intake × dietary crude protein content / 6.25 (Eq. 3). Total N excretion was computed as: total N intake - total Pd / 6.25 (Eq. 4). Total N deposition (Nd) was computed as: total Pd / 6.25 (Eq. 5). Total Pd was calculated using the equations:

6 $$ \begin{array}{l}\mathrm{q} = \left({\mathrm{D}}_{\mathrm{t} = \mathrm{n}}\hbox{--}\ {\mathrm{D}}_{\mathrm{t} = 0}\right) \times \mathrm{d}\mathrm{t}\\ {}\mathrm{Q} = \kern0.5em {\displaystyle \sum_{\mathrm{t}=0}^{\mathrm{t}=\mathrm{n}}\mathrm{q}}\end{array} $$

where:

D _t = n_: daily Pd on the final day of the trial, g/d
D _t = 0_: daily Pd on the initial day of the trial, g/d
Q: total Pd from the initial to the final trial day, g.

Average daily N excretion or deposition was determined as total N excretion or deposition divided by trial days (28d).

### Statistical analysis

All data were analyzed using the 2 × 2 factorial GLM procedure of SAS for a randomized complete block design (SAS 9.1). The model included the fixed effect of dietary protein source, protein level, associated two-way interactions and the random errors of a pen or a pig. Data were presented as the least square means and pooled SEM. The pen was used as the experimental unit for analyzing performance, total Pd, daily N deposition and excretion, and N efficiency data. Gilts were selected for slaughter and sampled individually, and therefore, the individual gilt was the experimental unit for carcass characteristics and meat quality, muscle AA profile, and SUN content. The minimum significance level considered was *P* < 0.05, while tendency was declared at 0.05 < *P* ≤ 0.10.

## Results

### Growth performance

Gilts offered CSM diets consumed more feed than those offered SBM diets (*P* = 0.03). Average daily gain tended to be greater in gilts offered CSM diets (*P* = 0.08). Average daily feed intake tended to increase in gilts offered reduced protein diets (*P* = 0.09). However, gain to feed ratio (G:F) was not altered by dietary treatments, and no interaction between dietary protein sources and levels for growth performance (*P* > 0.10) was observed (Table 2).

**Table 2:** Effect of dietary crude protein source and level on growth performance, protein deposition, and N balance of finishing gilts (90–113 kg, n = 6)

Item	Protein source		Protein level		SEM	*P*		
	Cottonseed meal	Soybean meal	12 %	14 %		Source	Level	Source × Level
Initial body weight, kg	90.30	88.80	90.30	88.70	1.86	0.58	0.56	0.93
Final body weight, kg	115.40	112.30	114.50	113.10	1.87	0.16	0.48	0.90
Average daily feed intake, kg/d	3.04	2.87	3.02	2.89	0.05	0.03	0.09	0.11
Gain-to-feed ratio	0.29	0.28	0.28	0.29	0.01	0.30	0.63	0.77
Average daily gain, kg/d	0.90	0.81	0.86	0.85	0.03	0.08	0.80	0.66
Total protein deposition^a^, kg	17.90	17.30	17.70	17.50	0.26	0.15	0.48	0.90
Serum urea nitrogen, mmol/L	7.54	7.19	6.50	8.23	0.45	0.59	0.01	0.24
Daily N intake, g/d	64.63	61.18	58.28	67.54	1.15	0.04	<0.01	0.91
Daily N deposition, g/d	20.43	20.87	20.53	20.76	0.22	0.17	0.47	0.91
Daily N excretion, g/d	44.20	40.30	37.70	46.8	1.31	0.05	<0.01	0.93
N efficiency^b^, kg/kg	0.32	0.35	0.35	0.31	0.01	0.06	<0.01	0.90

^a^Calculated from Eq.2 and Eq.6

^b^Computed as (daily N deposition / daily N intake)

### Protein deposition and N balance

There was no effect of dietary treatments on total Pd and daily Nd (*P* > 0.10) (Table 2). Gilts offered CSM diets had significantly greater daily N intake (*P* = 0.04) and N excretion (*P* = 0.05) than those offered SBM diet, but SUN content and N efficiency remained unchanged (*P* > 0.10). Daily N intake, N excretion, and SUN content were decreased in gilts offered reduced protein diets compared with those offered high protein diets (*P* < 0.05). N efficiency was simultaneously improved (*P* < 0.01). No dietary protein source × level interaction was observed on either Pd or N balance (*P* > 0.10).

### Carcass characteristics and meat quality

Neither dietary protein sources nor CP levels altered carcass characteristics, including carcass weight, dressing percentage, back fat depth, and LMA, throughout the duration of the experiment (*P* > 0.10) (Table 3).

**Table 3:** Effect of dietary crude protein source and level on carcass characteristics and meat quality of finishing gilts (90–113 kg, n = 6)

Item	Protein source		Protein level		SEM	*P*		
	Cottonseed meal	Soybean meal	12 %	14 %		Source	Level	Source × Level
Carcass traits								
Carcass weight, kg	86.80	85.70	87.40	85.00	1.80	0.66	0.36	0.51
Dressing percentage, %	75.65	76.60	76.74	75.50	0.63	0.30	0.19	0.22
Backfat depth (at the 10th rib), mm	26.15	25.08	25.08	26.19	1.12	0.52	0.52	0.82
Backfat depth (at the last rib), mm	26.16	24.50	25.56	25.10	1.88	0.39	0.81	0.23
*Longissimus* muscle area, cm^2^	50.80	51.90	50.50	52.20	1.46	0.60	0.43	0.89
Meat color								
L* (lightness)	44.03	45.92	45.15	44.80	0.79	0.10	0.76	0.62
a* (redness)	15.95	15.44	15.88	15.51	0.27	0.20	0.35	0.47
b* (yellowness)	2.98	3.20	3.09	3.09	0.29	0.60	0.98	0.68
pH								
pH_45min_^a^	5.83	5.95	5.73	6.06	0.11	0.48	0.04	0.84
pH_24h_^b^	5.48	5.47	5.46	5.48	0.02	0.60	0.48	0.76
Shear force, kg	1.51	1.52	1.54	1.48	0.08	0.93	0.58	0.25
Drip loss, %	2.03	2.45	2.24	2.24	0.29	0.33	0.99	0.64
Cooking loss, %	27.99	27.57	27.71	27.85	1.13	0.72	0.91	0.18
Marbling score (1–10 scale)	1.30	1.00	1.20	1.10	0.13	0.14	0.83	0.83
Intramuscular fat, %	2.84	2.62	2.37	3.09	0.46	0.65	0.14	0.71

^a^pH_45min_ = pH 45 minutes after postmortem

^b^pH_24h_ = pH 24 hours after postmortem

Except that pH_45min_ of fresh pork was slightly decreased in gilts fed 12 % CP diets than that in gilts fed 14 % CP diets (*P* < 0.05). Meat quality indicators, including meat color, pH_24h_ value, shear force, drip loss, cooking loss, marbling score and IMF content were not altered by dietary protein sources nor levels (*P* > 0.10) (Table 3). In addition, there was no interaction between dietary protein source and CP level on carcass characteristics and meat quality (*P* > 0.10).

### Amino acid profile of *longissmus dorsi* muscle

Dietary protein source rather than protein level altered amino acid profile of the LM. Besides the concentrations of phenylalanine (*P* = 0.01), tryptophan (*P* = 0.01), cysteine (*P* = 0.05) and tyrosine (*P* = 0.03) in the muscle were significantly reduced by CSM diets, and the concentrations of histidine, threonine, aspartic acid, glutamine, and serine were also tended to decreased (*P* < 0.10). We did not observe the interaction between dietary protein source and level on amino acid profile of the LM (*P* > 0.10) (Table 4).

**Table 4:** Effect of dietary crude protein source and level on amino acid profile of the *longissimus dorsi* muscle of finishing gilts (90–113 kg, n = 6), DM g/100 g

Item	Protein source		Protein level		SEM	*P*		
	Cottonseed meal	Soybean meal	12 %	14 %		Source	Level	Source × Level
Arginine	5.06	5.24	5.14	5.16	0.09	0.19	0.88	0.76
Histidine	3.74	3.96	3.84	3.85	0.09	0.10	0.93	0.42
Isoleucine	3.97	4.13	4.04	4.06	0.08	0.20	0.85	1.00
Leucine	6.83	7.16	6.98	7.02	0.14	0.11	0.85	0.79
Lysine	7.11	7.16	7.11	7.13	0.14	0.16	0.92	0.66
Methionine	2.26	2.33	2.25	2.33	0.04	0.23	0.17	0.59
Phenylalanine	3.40	3.70	3.53	3.57	0.08	0.01	0.69	0.55
Threonine	3.82	4.01	3.92	3.91	0.07	0.08	0.92	0.58
Tryptophan	0.86	0.91	0.88	0.89	0.01	<0.01	0.41	0.21
Valine	4.29	4.47	4.39	4.37	0.09	0.17	0.88	0.74
Alanine	4.85	5.04	4.96	4.93	0.09	0.15	0.79	0.63
Asparagine	7.51	7.90	7.74	7.68	0.15	0.08	0.76	0.54
Cysteine	0.87	0.92	0.89	0.90	0.01	0.05	0.42	0.53
Glutamate	11.69	12.26	12.00	11.95	0.23	0.09	0.88	0.52
Glycine	3.55	3.62	3.62	3.54	0.06	0.41	0.38	0.52
Proline	3.23	3.29	3.24	3.29	0.06	0.52	0.57	0.25
Serine	3.14	3.30	3.23	3.21	0.06	0.08	0.83	0.51
Tyrosine	2.52	2.71	2.60	2.63	0.06	0.03	0.67	0.65
Sulfur-containing amino acids^a^	3.13	3.24	3.14	3.24	0.05	0.15	0.21	0.57
Total amino acids	78.58	82.23	80.36	80.45	1.49	0.10	0.97	0.60

^a^Sum of methionine and cysteine

In addition, the ratio of phenylalanine to lysine in the LM was significantly decreased in gilts offered CSM diets compared with those offered SBM diets (*P* = 0.05). Similarly, the ratio of tyrosine to lysine also tended to decrease (*P* = 0.06) (Additional file 1: Table S1).

However, the ratios of AA to lysine in the LM were not altered by dietary protein levels (*P* > 0.10). Similarly, except tryptophan (*P* = 0.03), we did not observed any interaction between dietary protein source and level on the ratios of AA to lysine (*P* > 0.10).

### Intracellular concentrations of free amino acids in *longissimus dorsi* muscle

Gilts offered CSM diets had a greater free valine concentration in the LM compared with gilts offered SBM diets (*P* = 0.03) (Table 5). Also, the dietary protein source × level interaction for free valine concentration was observed (*P* < 0.01). Concentrations of free cysteine and serine also tended to increase in gilts which consumed CSM diets (*P* < 0.10). Additionally, the ratio of free methionine to lysine seemed to decrease in gilts offered CSM diets compared with those offered SBM diets (*P* = 0.06) (Additional file 2: Table S2).

**Table 5:** Effect of dietary crude protein source and level on intracellular free amino acid profile in the *longissimus dorsi* muscle of finishing gilts (90–113 kg, n = 6), μg/g

Item	Protein source		Protein level		SEM	*P*		
	Cottonseed meal	Soybean meal	12 %	14 %		Source	Level	Source × Level
Arginine	19.36	18.50	18.76	19.10	0.85	0.50	0.79	0.70
Histidine	84.49	84.66	84.69	84.46	1.43	0.93	0.91	0.79
Isoleucine	8.94	9.30	8.80	9.44	0.43	0.57	0.31	0.44
Leucine	15.70	15.03	16.07	14.67	0.52	0.71	0.09	0.24
Lysine	20.76	19.51	21.93	18.34	0.93	0.38	0.02	0.28
Methionine	5.26	5.51	5.73	5.05	0.18	0.34	0.02	0.44
Phenylalanine	10.94	11.03	11.33	10.64	0.28	0.84	0.11	0.48
Threonine	9.24	9.56	9.81	8.99	0.51	0.67	0.27	0.32
Tryptophan	2.92	3.02	2.82	3.12	0.12	0.58	0.09	0.48
Valine	14.05	12.36	13.15	13.26	0.49	0.03	0.88	<0.01
Alanine	158.09	165.34	161.15	162.48	6.40	0.42	0.89	0.21
Asparagine	5.37	5.13	5.58	4.92	0.31	0.59	0.15	0.35
Cysteine	0.61	0.29	0.58	0.33	0.12	0.08	0.12	0.53
γ-aminobutyric acid	0.36	0.36	0.37	0.36	0.01	0.99	0.50	0.66
Glutamate	12.11	13.63	12.31	13.43	1.03	0.31	0.45	0.82
Glutamine	92.04	83.21	100.73	74.53	10.01	0.52	0.08	0.56
Glycine	38.21	43.12	42.79	38.55	2.42	0.17	0.23	0.34
Proline	13.06	12.93	13.19	12.80	0.43	0.84	0.53	0.41
Serine	11.56	10.02	11.62	9.95	0.60	0.09	0.07	0.27
Tyrosine	11.11	10.77	10.97	10.90	0.46	0.62	0.95	0.70
Sulfur-containing amino acids^a^	5.87	5.80	6.30	5.37	0.23	0.84	0.01	0.78
Total amino acids	534.21	533.49	552.39	515.31	13.23	0.84	0.01	0.78

^a^Sum of methionine and cysteine

We found that reduced protein diets increased the intracellular concentrations of free lysine, methionine, SAA, and total amino acid (TAA) in the LM (*P* < 0.05), and tended to increase the concentrations of free leucine, glutamine, and serine (*P* < 0.10). However, free tryptophan concentration in the LM tended to decrease in gilts offered reduced protein diets compared with those offered high CP diets (*P* = 0.09) (Table 5).

The ratios of free arginine, histidine, isoleucine, tryptophan, valine, alanine, glutamine, tyrosine, EAA, and TAA to free lysine were decreased in the LM in gilts offered reduced protein diets relative to gilts offered high CP diets (*P* < 0.05) (Additional file 2: Table S2).

For AA profile of the LM, we found the relative ratio of free methionine to lysine was significantly decreased by CSM diets relative to SBM diets (*P* = 0.05). Meanwhile, the relative ratios of FAA, including arginine, histidine, isoleucine, tryptophan, valine, alanine, and tyrosine to lysine was significantly decreased by reduced protein diets (*P* < 0.05) (Table 6). It is interesting that the divergence order of ratios of FAA to free lysine of was subsequently cysteine > glutamate > SAA > isoleucine > leucine > threonine and methionine (Table 6).

**Table 6:** Effect of dietary protein source and level on relative ratios of free amino acids to lysine in the *longissimus dorsi* muscle standardized by the muscle entire amino acid profile of finishing gilts (90–113 kg, n = 6)

Item	Protein source		Protein level		SEM	*P*		
	Cottonseed meal	Soybean meal	12 %	14 %		Source	Level	Source × Level
Arginine	1.31	1.33	1.20	1.44	0.04	0.82	<0.01	0.50
Histidine	7.87	8.14	7.32	8.69	0.37	0.62	0.02	0.82
Isoleucine	0.78	0.86	0.72	0.92	0.05	0.27	0.01	0.93
Leucine	0.79	0.79	0.76	0.82	0.03	0.84	0.16	0.94
Lysine	1.00	1.00	1.00	1.00	0.00	-	-	-
Methionine	0.80	0.89	0.84	0.85	0.03	0.05	0.87	0.13
Phenylalanine	1.11	1.13	1.07	1.18	0.05	0.81	0.12	0.94
Threonine	0.82	0.90	0.82	0.90	0.04	0.21	0.19	0.68
Tryptophan	1.19	1.26	1.07	1.38	0.06	0.45	<0.01	0.61
Valine	1.13	1.06	0.99	1.20	0.05	0.36	0.01	0.17
Alanine	11.40	12.47	10.78	13.10	0.65	0.26	0.02	0.19
Cysteine	0.24	0.12	0.22	0.14	0.05	0.10	0.25	0.64
Glutamate	0.36	0.42	0.33	0.44	0.04	0.23	0.04	0.65
Glycine	3.73	4.54	3.91	4.36	0.33	0.11	0.36	0.38
Proline	1.40	1.51	1.35	1.56	0.10	0.45	0.15	0.75
Serine	1.26	1.15	1.18	1.23	0.08	0.35	0.65	0.77
Tyrosine	1.52	1.50	1.40	1.63	0.07	0.83	0.03	0.76
Sulfur-containing amino acids^a^	0.64	0.67	0.67	0.65	0.03	0.49	0.72	0.36
Essential amino acids	1.61	1.65	1.52	1.74	0.06	0.58	0.01	0.86
Non-essential amino acids	2.28	2.50	2.18	2.59	0.12	0.23	0.03	0.26
Total amino acids	1.93	2.05	1.83	2.15	0.08	0.30	0.02	0.38

^a^Sum of methionine and cysteine.

## Discussion

Global intensive demand for SBM leads to a rapid rising of soybean price, which aggravates the feeding cost of pigs. On the other hand, nitrogen excretion from pigs needs to be minimized due to environmental concerns. Therefore, there is a critical need to find an economical and reliable alternative for SBM without impairing the efficiency of nitrogen utilization.

Knabe et al. [22] supposed that feed intake did not differ in growing pigs fed either CSM or SBM diets, when free gossypol content in CSM was reduced to a level without affecting pig performance. The diets with ≤ 4 % reduction of CP, in which crystalline AA were added to meet EAA requirement, did not impede the performance of finishing pigs [5, 23–28]. In this study, our data strongly supported that pig performance was not depressed when pigs were fed either CSM diets or diets with a certain reduction of CP as long as SID EAA and available energy were maintained to meet nutrition requirements of pigs.

In modern livestock production, producers wish to increase N efficiency and reduce N excretion because nitrogen compounds are important contributors to greenhouse gas emissions [29]. Accumulating evidence demonstrated that reduced CP diets improved N efficiency in pigs [24, 27, 30, 31]. The results of the current study were consistent with previous studies. On the other hand, to our knowledge, very limited data are available on comparison of N balance between pigs fed CSM and SBM diets until now. We found that CSM diets did not alter body protein deposition, SUN, Nd, and N efficiency in finishing gilts, even though daily N intake was slightly increased by 5.6 %, which is consistent with a previous study [32].

In previous studies, digestibility of CSM was found to be lower than that of SBM [2, 23, 33], which could be due to the relative low quality and high gossypol content in CSM used in these previous studies. However, as CSM quality and gossypol content were emphasized in cotton seeds oil manufacture, for example, the CSM used in the present study provides 46.09 % CP and contains only 63 mg/kg of free gossypol based on feed weight, the difference in digestibility of AA between CSM and SBM becomes minor.

Based on our knowledge, there were very limited data comparing carcass characteristics and meat quality of pigs fed CSM and SBM diet. In this study, carcass characteristics and meat quality did not alter when CSM was used to replace SBM. Similarly, Szabo et al. [34] reported that IMF content, drip loss, and carcass pH value were not altered in pigs fed a diet replacing SBM with sunflower meal, ground peas, or fish meal. Consistently, we did not observe that the replacement of SBM by CSM in diets affect carcass characteristics and meat quality of finishing gilts.

Previous studies reported that dietary protein contents have limited effects on carcass traits and meat quality characteristics [6, 26, 35, 36] when SID EAA and energy were maintained to meet nutrition requirements in diets. In the present study, we also showed that dietary protein level did not affect carcass characteristics and meat quality, except that pH_45min_ of fresh pork was decreased by 5.4 % when gilts offered reduced protein diets. Additionally, it has been observed that feeding pigs a low protein diet resulted in greater back fat depth and IMF content, and more abundant in marbling in both *longissimus dorsi* and *semimembranosus* muscle [29, 35], which was not observed in the present study. The possible reason could be due to the feed composition and the balance of AA profile.

There are very limited data available on effect of dietary protein on muscle AA profile in finishing pigs. In the present study, we reported the concentrations of phenylalanine, tryptophan, cysteine and tyrosine of the LM decreased in gilts offered CSM diets. However, muscle AA profile was not altered by dietary protein level.

Furthermore, skeletal muscle is the largest reservoir of FAA in the body, which contains approximately 75 % of the entire FAA pool of the body [37, 38]. The size and composition of the FAA pool depends on several processes, including availability of circulating FAA, an increased AA circulation between skeletal muscle and other tissues resulting from AA utilization (e.g., by protein synthesis) or protein catabolism [39, 40].

The pigs fed reduced protein diets typically decrease their circulative AA pool size than the pigs fed high protein diets [5]. It has been shown that the variation of total SAA supplies or the hypocaloric dietary treatment could affect the AA composition of muscles [41, 42]. Thus, in the current study, it is reasonable that dietary protein source altered the concentrations of LM AA, including phenylalanine, tryptophan and cysteine. On the other hand, there are very few reports regarding the nutritional effect on free AA profile of muscles. In the present study, we discovered that feeding reduced protein diets enlarged free FAA pool of the LM in pigs. We deduced that, it may be due to unbalanced AA supplies in the reduced protein diets, even though all essential AA were balanced by supplementation of crystalline AA based on the latest edition of NRC [8], which may result in an increased protein catabolism or more intensive protein turnover in muscle beyond our detection in present study, although its biological meaning needs further exploration.

We also noticed that the ratios of FAA to free lysine is subsequently cysteine > glutamate > SAA > isoleucine > leucine > threonine and methionine, which could be the limiting factors negatively affecting muscle protein synthesis and muscle hypertrophy [43]. Even though intracellular concentration of FAA in muscle is below three orders of magnitude than muscle AA concentrations, they may take an important role in muscle protein synthesis and turnover, which warrant further study.

## Conclusions

It is feasible to use CSM as a main protein source to replace SBM completely in terms of pig growth performance, carcass characteristics and meat quality, as well as dietary N efficiency, as long as SID EAA and ME were maintained to meet nutrition requirements of pigs. Our study reported a novel finding that, the substitution of SBM by CSM increased the concentrations of phenylalanine, tryptophan, cysteine, and tyrosine in muscle, as well as intracellular concentration of free valine in muscle. Furthermore, reducing protein diets enlarged muscle intracellular concentrations of specific FAA, and shrank the ratio of specific FAA to lysine of pigs.

## Additional files

Additional file 1: Table S1. Effect of dietary crude protein source and level on the ratio of AA to lysine in *longissimus dorsi* muscle of finishing (90-113 kg) gilts (n=6). (DOCX 20 kb) Additional file 2: Table S2. Effect of dietary crude protein source and level on the ratio of intracellular FAA to free lysine in *longissimus dorsi* muscle of finishing (90-113 kg) gilts (n=6). (DOCX 19 kb)

## Abbreviations

AA: amino acid
BW: body weight
CP: crude protein
CSM: cottonseed meal
EAA: essential amino acid
FAA: free amino acid
G:F: gain to feed ratio
HCW: hot carcass weight
IMF: intramuscular fat content
LM: *longissimus* muscle
LMA: *longissimus* muscle area
ME: metabolizable energy
Nd: N deposition
Pd: protein deposition
SAA: sulfur-containing amino acid
SBM: soybean meal
SID: standardized ileal digestibility
SUN: serum urea nitrogen
STTD: standardized total tract digestible
TAA: total amino acid

## References

[1] Gill M, Smith P, Wilkinson J (2010). Mitigating climate change: the role of domestic livestock. Animal.

[2] González-Vega J, Stein HH (2012). Amino acid digestibility in canola-, cottonseed-and sunflower-products fed to finishing pigs. J Anim Sci.

[3] Withers WA, Carruth FE (1915). Gossypol, the toxic substance in cottonseed meal. J Agric Res.

[4] Zhang WJ, Xu ZR, Zhao SH, Sun JY, Yang X (2007). Development of a microbial fermentation process for detoxification of gossypol in cottonseed meal. Anim Feed Sci Tech.

[5] Figueroa JL, Lewis AJ, Miller PS, Fischer RL, Gómez RS, Diedrichsen RM (2002). Nitrogen metabolism and growth performance of gilts fed standard corn-soybean meal diets or low-crude protein, amino acid-supplemented diets. J Anim Sci.

[6] Madrid J, Martínez S, López C, Orengo J, López MJ, Hernández F (2013). Effects of low protein diets on growth performance, carcass traits and ammonia emission of barrows and gilts. Anim Prod Sci.

[7] GB13078-2001. Hygienical standard for feed. National Standards of the People’s Republic of China; 2001.

[8] NRC (2012). Nutrient requirements of swine.

[9] Huff-Lonergan E, Baas TJ, Malek M, Dekkers JC, Prusa K, Rothschild MF (2002). Correlations among selected pork quality traits. J Anim Sci.

[10] Jia J, Schinckel AP, Forrest JC, Chen W, Wagner JR (2010). Prediction of lean and fat composition in swine carcasses from ham area measurements with image analysis. Meat Sci.

[11] NPPC (1999). Oficial color and marbling standards.

[12] Aaslyng MD, Bejerholm C, Ertbjerg P, Bertram HC, Andersen HJ (2003). Cooking loss and juiciness of pork in relation to raw meat quality and cooking procedure. Food Qual Prefer.

[13] Straadt IK, Rasmussen M, Andersen HJ, Bertram HC (2007). Aging-induced changes in microstructure and water distribution in fresh and cooked pork in relation to water-holding capacity and cooking loss-A combined confocal laser scanning microscopy (CLSM) and low-field nuclear magnetic resonance relaxation study. Meat Sci.

[14] Ciobanu DC, Bastiaansen JWM, Lonergan SM, Thomsen H, Dekkers JC, Plastow GS (2004). New alleles in calpastatin gene are associated with meat quality traits in pigs. J Anim Sci.

[15] Van Oeckel MJ, Warnants N, Boucqué CV (1999). Pork tenderness estimation by taste panel, Warner-Bratzler shear force and on-line methods. Meat Sci.

[16] Zhang JX, Yin JD, Zhou X, Li FN, Ni JJ, Dong B (2008). Effects of lower dietary lysine and energy content on carcass characteristics and meat quality in growing-finishing pigs. Asian-Austr J Anim Sci.

[17] AOAC (2007). Official methods of analysis.

[18] Hron R, Kuk M, Abraham G (1990). Determination of free and total gossypol by high performance liquid chromatography. J Am Oil Chem Soc.

[19] Heinrikson RL, Meredith SC (1984). Amino acid analysis by reverse-phase high-performance liquid chromatography: precolumn derivatization with phenylisothiocyanate. Anal Biochem.

[20] Serra X, Gil F, Pérez-Enciso M, Oliver MA, Vázquez JM, Gispert M (1998). A comparison of carcass, meat quality and histochemical characteristics of Iberian (Guadyerbas line) and Landrace pigs. Livest Prod Sci.

[21] Li F, Duan Y, Li Y, Tang Y, Geng M, Oladele OA (2015). Effects of dietary n-6: n-3 PUFA ratio on fatty acid composition, free amino acid profile and gene expression of transporters in finishing pigs. Brit J Nutr.

[22] Knabe D, Tanksley T, Hesby J (1979). Effect of lysine, crude fiber and free gossypol in cottonseed meal on the performance of growing pigs. J Anim Sci.

[23] Batterham E, Andersen L, Baigent D, Darnell R, Taverner M (1990). A comparison of the availability and ileal digestibility of lysine in cottonseed and soya-bean meals for grower/finisher pigs. Brit J Nutr.

[24] Canh TT, Aarnink AJA, Schutte JB, Sutton A, Langhout DJ, Verstegen MWA (1998). Dietary protein affects nitrogen excretion and ammonia emission from slurry of growing-finishing pigs. Livest Prod Sci.

[25] Knowles T, Southern L, Bidner T, Kerr B, Friesen K (1998). Effect of dietary fiber or fat in low-crude protein, crystalline amino acid-supplemented diets for finishing pigs. J Anim Sci.

[26] Figueroa JL, Lewis AJ, Miller PS, Fischer RL, Diedrichsen RM (2003). Growth, carcass traits, and plasma amino acid concentrations of gilts fed low-protein diets supplemented with amino acids including histidine, isoleucine, and valine. J Anim Sci.

[27] Galassi G, Colombini S, Malagutti L, Crovetto G, Rapetti L (2010). Effects of high fibre and low protein diets on performance, digestibility, nitrogen excretion and ammonia emission in the heavy pig. Anim Feed Sci Tech.

[28] Gloaguen M, Le Floc’h N, Corrent E, Primot Y, van Milgen J (2014). The use of free amino acids allows formulating very low crude protein diets for piglets. J Anim Sci.

[29] Wood JD, Lambe NR, Walling GA, Whitney H, Jagger S, Fullarton PJ (2013). Effects of low protein diets on pigs with a lean genotype. 1. Carcass composition measured by dissection and muscle fatty acid composition. Meat Sci.

[30] Kerr B, Easter R (1995). Effect of feeding reduced protein, amino acid-supplemented diets on nitrogen and energy balance in grower pigs. J Anim Sci.

[31] O’Connell J, Callan J, O’Doherty J (2006). The effect of dietary crude protein level, cereal type and exogenous enzyme supplementation on nutrient digestibility, nitrogen excretion, faecal volatile fatty acid concentration and ammonia emissions from pigs. Anim Feed Sci Tech.

[32] Prawirodigdo S, Batterham E, Andersen L, Dunshea F, Farrell D (1997). Nitrogen retention in pigs given diets containing cottonseed meal or soybean meal. Anim Feed Sci Tech.

[33] Tanksley T, Knabe D, Purser K, Zebrowska T, Corley J (1981). Apparent digestibility of amino acids and nitrogen in three cottonseed meals and one soybean meal. J Anim Sci.

[34] Szabo C, Jansman A, Babinszky L, Kanis E, Verstegen M (2001). Effect of dietary protein source and lysine: DE ratio on growth performance, meat quality, and body composition of growing-finishing pigs. J Anim Sci.

[35] Kerr B, McKeith F, Easter R (1995). Effect on performance and carcass characteristics of nursery to finisher pigs fed reduced crude protein, amino acid-supplemented diets. J Anim Sci.

[36] Teye GA, Sheard PR, Whittington FM, Nute GR, Stewart A, Wood JD (2006). Influence of dietary oils and protein level on pork quality. 1. Effects on muscle fatty acid composition, carcass, meat and eating quality. Meat Sci.

[37] Graham TE, Sgro V, Friars D, Gibala MJ (2000). Glutamate ingestion: the plasma and muscle free amino acid pools of resting humans. Am J Physiol-Endoc M.

[38] Davis TA, Fiorotto ML (2009). Regulation of muscle growth in neonates. Curr Opin Clin Nutr Metab Care.

[39] Proud CG (2004). mTOR-mediated regulation of translation factors by amino acids. Biochem Bioph Res Co.

[40] Hundal HS, Taylor PM (2009). Amino acid transceptors: gate keepers of nutrient exchange and regulators of nutrient signaling. Am J Physiol-Endoc M.

[41] Conde-Aguilera JA, Lefaucheur L, Tesseraud S, Mercier Y, Le Floc’h N, van Milgen J (2015). Skeletal muscles respond differently when piglets are offered a diet 30 % deficient in total sulfur amino acid for 10 days. Eur J Nutr.

[42] David MR, Paul MW, Lawrence AL, Anders AS, Wayne KT, Donald AM (1984). Metabolic and structural changes in skeletal muscle during hypocaloric dieting. Am J Clin Nuir.

[43] Sales F, Pacheco D, Blair H, Kenyon P, McCoard S (2013). Muscle free amino acid profiles are related to differences in skeletal muscle growth between single and twin ovine fetuses near term. SpringerPlus.

