# Supplementary material for: Influences of dietary protein sources and crude protein levels on intracellular free amino acid profile in the longissimus dorsi muscle of finishing gilts
Source: J Anim Sci Biotechnol. 2015 Dec 18;6:52. doi: 10.1186/s40104-015-0052-x (PMC4683754; doi:10.1186/s40104-015-0052-x)
Supplement: Additional file 1: Table S1. — Effect of dietary crude protein source and level on the ratio of AA to lysine in longissimus dorsi muscle of finishing (90-113 kg) gilts (n=6). (DOCX 20 kb) [file 40104_2015_52_MOESM1_ESM.docx]

Table S1. Effect of dietary crude protein source and level on the ratio of AA to lysine in *longissimus dorsi* muscle of finishing (90-113 kg) gilts (n=6)

| Item | Protein source | |  | Protein level | | SEM | *P* | | |
| --- | --- | --- | --- | --- | --- | --- | --- | --- | --- |
|  | Cottonseed meal | Soybean meal |  | 12% | 14% |  | Source | Level | Source × Level |
| Arginine | 0.72 | 0.72 |  | 0.72 | 0.72 | 0.00 | 0.48 | 0.59 | 0.49 |
| Histidine | 0.54 | 0.54 |  | 0.54 | 0.54 | 0.01 | 0.21 | 0.91 | 0.21 |
| Isoleucine | 0.57 | 0.57 |  | 0.57 | 0.57 | 0.00 | 0.70 | 0.51 | 0.16 |
| Leucine | 0.98 | 0.99 |  | 0.98 | 0.98 | 0.00 | 0.39 | 0.72 | 0.36 |
| Lysine | 1.00 | 1.00 |  | 1.00 | 1.00 | 0.00 | - | - | - |
| Methionine | 0.32 | 0.32 |  | 0.32 | 0.33 | 0.00 | 1.00 | 0.14 | 0.82 |
| Phenylalanine | 0.49 | 0.51 |  | 0.50 | 0.50 | 0.01 | 0.05 | 0.64 | 0.53 |
| Threonine | 0.55 | 0.55 |  | 0.55 | 0.55 | 0.00 | 0.18 | 0.88 | 0.39 |
| Tryptophan | 0.12 | 0.13 |  | 0.12 | 0.13 | 0.00 | 0.62 | 0.62 | 0.03 |
| Valine | 0.61 | 0.62 |  | 0.62 | 0.61 | 0.00 | 0.71 | 0.33 | 0.52 |
| Alanine | 0.70 | 0.69 |  | 0.70 | 0.69 | 0.00 | 0.69 | 0.24 | 0.90 |
| Asparagine | 1.08 | 1.09 |  | 1.09 | 1.08 | 0.01 | 0.19 | 0.28 | 0.45 |
| Cysteine | 0.13 | 0.13 |  | 0.13 | 0.13 | 0.00 | 0.88 | 0.88 | 0.56 |
| Glutamate | 1.68 | 1.69 |  | 1.69 | 1.68 | 0.01 | 0.33 | 0.34 | 0.48 |
| Glycine | 0.51 | 0.50 |  | 0.51 | 0.50 | 0.01 | 0.37 | 0.23 | 0.70 |
| Proline | 0.46 | 0.45 |  | 0.45 | 0.46 | 0.01 | 0.35 | 0.49 | 0.58 |
| Serine | 0.45 | 0.46 |  | 0.45 | 0.45 | 0.00 | 0.30 | 0.53 | 0.30 |
| Tyrosine | 0.36 | 0.37 |  | 0.36 | 0.37 | 0.00 | 0.06 | 0.44 | 0.77 |
| Sulfur-containing amino acids ^1^ | 0.45 | 0.45 |  | 0.44 | 0.45 | 0.01 | 0.71 | 0.10 | 0.74 |
| Essential amino acids | 5.90 | 5.95 |  | 5.91 | 5.93 | 0.02 | 0.23 | 0.60 | 0.94 |
| Non-essential amino acids | 5.36 | 5.37 |  | 5.38 | 5.39 | 0.03 | 0.75 | 0.56 | 0.48 |
| Total amino acids | 11.26 | 11.32 |  | 11.29 | 11.29 | 0.06 | 0.47 | 0.91 | 0.73 |

^1^ Sum of methionine and cysteine.
