# Supplementary material for: Influences of dietary protein sources and crude protein levels on intracellular free amino acid profile in the longissimus dorsi muscle of finishing gilts
Source: J Anim Sci Biotechnol. 2015 Dec 18;6:52. doi: 10.1186/s40104-015-0052-x (PMC4683754; doi:10.1186/s40104-015-0052-x)
Supplement: Additional file 2: Table S2. — Effect of dietary crude protein source and level on the ratio of intracellular FAA to free lysine in longissimus dorsi muscle of finishing (90-113 kg) gilts (n=6). (DOCX 19 kb) [file 40104_2015_52_MOESM2_ESM.docx]

Table S2. Effect of dietary crude protein source and level on the ratio of intracellular FAA to free lysine in *longissimus dorsi* muscle of finishing (90-113 kg) gilts (n=6)

| Item | Protein source | |  | Protein level | | SEM | *P* | | |
| --- | --- | --- | --- | --- | --- | --- | --- | --- | --- |
|  | Cottonseed meal | Soybean meal |  | 12% | 14% |  | Source | Level | Source × Level |
| Arginine | 0.95 | 0.96 |  | 0.87 | 1.05 | 0.03 | 0.89 | < 0.01 | 0.48 |
| Histidine | 0.42 | 0.43 |  | 3.96 | 4.70 | 0.22 | 0.50 | 0.02 | 0.70 |
| Isoleucine | 0.44 | 0.49 |  | 0.41 | 0.52 | 0.03 | 0.27 | < 0.01 | 0.82 |
| Leucine | 0.77 | 0.78 |  | 0.74 | 0.81 | 0.03 | 0.73 | 0.14 | 0.92 |
| Lysine | 1.00 | 1.00 |  | 1.00 | 1.00 | 0.00 | - | - | - |
| Methionine | 0.26 | 0.29 |  | 0.27 | 0.28 | 0.01 | 0.06 | 0.50 | 0.15 |
| Phenylalanine | 0.54 | 0.58 |  | 0.53 | 0.59 | 0.03 | 0.34 | 0.09 | 0.81 |
| Threonine | 0.45 | 0.50 |  | 0.45 | 0.49 | 0.02 | 0.15 | 0.21 | 0.71 |
| Tryptophan | 0.15 | 0.16 |  | 0.13 | 0.17 | 0.01 | 0.32 | < 0.01 | 0.90 |
| Valine | 0.69 | 0.65 |  | 0.61 | 0.73 | 0.03 | 0.35 | 0.02 | 0.16 |
| Alanine | 7.91 | 8.64 |  | 7.50 | 9.05 | 0.44 | 0.26 | 0.02 | 0.17 |
| Cysteine | 0.03 | 0.02 |  | 0.03 | 0.02 | 0.01 | 0.15 | 0.37 | 0.75 |
| Glutamate | 0.60 | 0.71 |  | 0.56 | 0.75 | 0.06 | 0.22 | 0.05 | 0.61 |
| Glycine | 1.90 | .226 |  | 1.98 | 2.17 | 0.17 | 0.16 | 0.44 | 0.31 |
| Proline | 0.65 | 0.68 |  | 0.61 | 0.72 | 0.04 | 0.56 | 0.08 | 0.96 |
| Serine | 0.57 | 0.52 |  | 0.53 | 0.56 | 0.04 | 0.39 | 0.67 | 0.81 |
| Tyrosine | 0.55 | 0.56 |  | 0.51 | 0.60 | 0.02 | 0.75 | 0.02 | 0.71 |
| Sulfur-containing amino acids ^1^ | 0.29 | 0.30 |  | 0.29 | 0.30 | 0.01 | 0.49 | 0.88 | 0.34 |
| Essential amino acids | 9.48 | 9.83 |  | 8.96 | 10.34 | 0.35 | 0.58 | 0.01 | 0.87 |
| Non-essential amino acids | 12.21 | 13.39 |  | 11.74 | 13.86 | 0.64 | 0.21 | 0.03 | 0.21 |
| Total amino acids | 21.69 | 23.21 |  | 20.70 | 24.20 | 0.94 | 0.27 | 0.02 | 0.36 |

^1^ Sum of methionine and cysteine.
